# Experiences of patients talking about mental illness with their children: a qualitative study

**DOI:** 10.3389/fpsyg.2024.1504130

**Published:** 2025-01-22

**Authors:** Elizabeth Rapa, Athif Ilyas, Simone de Cassan, Louise J. Dalton

**Affiliations:** ^1^Department of Psychiatry, Warneford Hospital, University of Oxford, Oxford, United Kingdom; ^2^Oxford Health NHS Foundation Trust, Oxford, United Kingdom

**Keywords:** mental illness, parents, children, families, communication, healthcare professionals

## Abstract

**Background:**

Many adults with mental illness have dependent children; these parents must navigate decisions about whether and what to tell their children about the illness. Parents are often influenced by shame and guilt about their disorder, and a desire to protect their children from distress. Communication about parental mental illness can have important benefits for children’s psychological outcomes; professionals could be central in facilitating these conversations. This study explored parents’ experiences of talking to children about their mental illness and the role of their clinical team in this process.

**Methods:**

Fifteen parents with a mental illness under NHS care in England participated in qualitative interviews. Audio recordings were transcribed verbatim and analyzed using an inductive coding approach following the principles of thematic analysis.

**Results:**

Thematic analysis identified 4 themes: 1. Factors that affect what children are told about parental mental illness, 2. Perceived benefits of talking to children about parental mental illness, 3. Experience of ‘who’ talks to children about parental mental illness, 4. Role of healthcare professionals in supporting families to talk about parental mental illness. There was wide variation in what information was shared with children, influenced by fears about how to share particular diagnoses and the impact of the information on parents themselves and their family. Participants reported that no professionals had asked them what their children knew about their mental illness or offered advice on how to have these conversations, but all would have welcomed this guidance.

**Conclusion:**

Improving communication about parental mental illness requires targeted training programs for professionals and age-appropriate resources for families. This study emphasizes the critical role of fostering effective communication about parental mental illness to enhance children’s mental health and strengthen family functioning.

## Introduction

Around the world 15–23% of children live with parent with a mental disorder (Leijdesdorff et al., 2017; Maybery and Reupert, 2018), with a recent UK-based study reporting that 22% of parents and caregivers screened positive for mental health problems (Grant et al., 2024). Furthermore, a UK cohort study reported that one in four children aged 0–16 years will have experienced maternal mental illness, and by the time a child reaches 16 years old the cumulative risk of maternal mental illness is over 50% (Abel et al., 2019).

Children who have a parent with a mental disorder are at increased risk of poorer outcomes including cognitive, social and emotional development, behavioral difficulties and disorganized attachment (Goodman et al., 2011; Stein and Harold, 2015; Scarlett et al., 2023) as well as being at a higher chance of developing a mental illness themselves (Mowbray et al., 2006; Powell et al., 2023).

Parental perspectives on whether, and to what extent, children should be informed about mental illness vary. Parents often feel shame and guilt about their illness (Gladstone et al., 2011); they want to protect children from the impact of their disorder and for them to have a ‘normal childhood’ (Nolte and Wren, 2016). They also express uncertainty about the benefits of telling their children, fearing too much information could be a burden (Stallard et al., 2004). Parents can underestimate how much young children understand or notice about parental illness (Stallard et al., 2004; Nolte and Wren, 2016), with research indicating that those as young as 5 were aware something was wrong, despite their parents’ attempts to conceal evidence of their mental health difficulties (Gladstone et al., 2011). Other studies have found that parents *do* want children to understand more about their mental illness and would like advice and support about how to have these conversations (Stallard et al., 2004). Parents hope this will rectify any misunderstandings about the illness and thereby reduce children’s sense of responsibility for causing the illness or for any family problems (Stallard et al., 2004; Gladstone et al., 2011). Other caregivers, such as the child’s ‘well’ parent or grandparent, are often tasked with deciding what children are told about parental mental illness, answering their questions and suggesting sources of information about the disorder (Ballal and Navaneetham, 2018). However, effective disclosure by these individuals is often lacking due to a desire to provide ‘reassurance’ and ‘protection’ to children through withholding information about parental mental illness (Ballal and Navaneetham, 2018).

There are documented challenges to talking about parental mental illness within the family (Van Parys and Rober, 2013; Van Parys et al., 2015). Children and adults with lived-childhood experience of parental depression have described these conversations as ‘taboo’ which inhibits asking questions (Van Parys et al., 2015). Mirroring parents’ own fears, children also want to protect their parents from distress about the situation and perceive that conversations may add further burden and stress to their family situation (Van Parys et al., 2015). Tackling these obstacles to communication is crucial, as evidence suggests a comprehensive understanding can help to mitigate the adverse consequences associated with parental mental illness (Mordoch and Hall, 2008). Information enables children to understand their parent’s behavior and their own experiences (Van Parys et al., 2015), which can improve psychological well-being (Grove et al., 2016). Family communication can help children develop the language skills to talk to others about the mental illness; this allows them to access support, as well as speak about parental difficulties without embarrassment (Wolpert et al., 2015). Interventions to promote family discussion about parental mental illness have shown positive effects on children’s feelings of worry and responsibility for their parent (Pihkala et al., 2012), children’s internalizing symptoms (Beardslee et al., 2003) and decreased shame (Templeton, 2012; Pihkala et al., 2017). Parents recognize the positive impact on their children, which in turn contributes to their own sense of ‘relief’ and improved family communication (Pihkala et al., 2012).

Professionals can play a key role in facilitating conversations about parental mental illness in families; children want support from mental healthcare professionals (Tabak et al., 2016) and to have opportunities to discuss their parent’s symptoms and behaviors with the team (Ostman, 2008). Furthermore, parents also want help from professionals about how to talk to their children (Nolte and Wren, 2016; Ballal and Navaneetham, 2018). Social and health professionals are in agreement regarding the benefits of psychoeducation for children about parental mental illness (Tapias et al., 2021). Nevertheless, a notable gap persists in the provision of specific support from professionals for families on how to talk to children. This may reflect the ‘invisibility’ of children to their parents’ mental healthcare providers (Meadus and Johnson, 2000; Gladstone et al., 2011; Wahl et al., 2017; Cudjoe and Chiu, 2020) and the common disconnect between children’s and adult services. A desire for further training remains a consistent theme in the wider drive towards family-focused practice (Oakes et al., 2023) and mental healthcare professionals’ self-identified needs to embed family communication within their clinical care (Dalton et al., 2024).

In order to be able to provide effective tools and strategies for promoting family communication, we need to fully understand the facilitators and obstacles for parents with mental illness about initiating conversations with their children. Therefore, the current study aimed to specifically explore parents’ experiences about talking to children about their mental illness and understand:

- The information conveyed by parents to their children.
- The rationales underlying the choice to disclose or withhold information about parental mental illness.
- The assistance provided by clinical teams in facilitating communication with their children.
- Aspects that would have been beneficial for parents in the communication process.

## Materials and methods

### Design

This exploratory qualitative study used semi-structured in-depth interviews and is reported in accordance with the Consolidated Criteria for Reporting Research checklist for qualitative research (Tong et al., 2007).

### Participants

Convenience and purposive sampling were used to invite 25 parents with mental illness from one NHS Healthcare Trust in the UK. Four individuals did not respond, five individuals declined to be interviewed, and one individual did not attend the interview; the remaining 15 individuals were interviewed.

Participants were eligible if they were under the care of a community mental health team with any diagnosed mental health disorder and had a child over the age of 2 years old (this being the age where children begin to form a mental image of their caregiver) (Stein et al., 2019).

### Study recruitment

Study adverts were developed by the research team and placed in community mental health waiting areas. Three authors (ER, AI, LD) attended community mental health team meetings and sent emails to staff (including the participant information sheet) to share with potential participants. Individuals who met eligibility criteria were approached by members of their clinical team either face to face, by telephone or email. Those who expressed an interest in the study either contacted the authors directly themselves or gave consent to the clinical team to share their email address or telephone number with the authors who then contacted them directly about the study. Once contact was established, individuals were provided with the participant information sheet which included the study rationale and the research team’s interest in this topic. Subsequently a date and time for the interview were agreed. Individuals were informed that participation was voluntary and that their clinical team would not be informed whether or not they chose to participate in the study. Written informed consent was obtained prior to the interview.

### Data collection

Semi-structured interviews were conducted between January and June 2023. A topic guide (Table 1) was developed and was informed by the study’s aims and objectives, pilot testing with clinicians in the research team, and prior experience of the study team.

**Table 1:** Topic guide.

**Topic area**
Context of participant’s family structure and experience of their mental health disorder
Exploration of participant’s experience of the impact of the mental health disorder on their role as a parent
Exploration of participant’s perception and experience of their children’s awareness and understanding of parental mental health disorder
Exploration of participant’s beliefs and behavior about communication with their children regarding parental mental health disorder
Factors that may have influenced communication during children’s lifetime
Participants’ perception of the needs for parents who have mental health disorders and how these could be addressed

Interviews were completed by two researchers, AI and LD, with the majority being conducted by AI. Interviews were completed when data saturation was reached, whereby no further categories were identified. All interviews were conducted in a private space via Microsoft Teams or telephone and audio recorded; no field notes were taken. Interviews lasted between 16 and 87 min (mean = 53 min); no non-participants were present during the interviews. Participants were not known to the interviewers. No repeat interviews were conducted.

### Data analysis

Audio-recordings were transcribed verbatim either by one of the authors, AI, or using a university approved transcription service. Transcripts were thereafter anonymized and checked for accuracy by the research team. Transcripts were not returned to participants for comment or correction. Analysis was guided by reflexive thematic analysis, which was chosen because its theoretically flexible approach allows for identification and thorough analysis of patterns across a qualitative dataset to offer compelling insights into the real word experiences of participants (Braun and Clarke, 2006, 2019).

AI, LD and ER read and re-read the transcripts to gain a sense of the story of each individual participant. Data were coded using Microsoft Excel (Office 365 version) and these codes were clustered into groups of similar meaning from which potential themes were identified. These potential themes were organized and further developed through mind-mapping activities and discussions within the research team. These discussions included a critical examination of any potential preconceived ideas or assumptions that may have influenced the data analysis. This whole process continued to proceed iteratively until the final themes were generated. Participants were not invited to provide feedback on the findings.

### Ethical statement

All participants were given written information about the study and provided informed consent before taking part in the study. Participants were aware of their right to withdraw at any stage; none withdrew from the study. Data protection procedures were followed. Ethical approvals were obtained from the National Health Service (NHS) Health Research Authority (Reference number: 21/WA/0345).

### Reflexivity, research group and context

Three authors (ER, SdC, LD) identify as white females and one author (AI) as an Asian male. ER and LD are associate professors who have conducted extensive research on parental mental health and have a specific interest in communication with children about serious illness; LD is also a consultant clinical psychologist and has worked extensively with parents and children within an NHS context. AI and SdC are clinical academic doctors who have experience of delivering clinical services to adults and children in NHS mental health settings; at the time of the study both were NIHR Academic Clinical Fellows in psychiatry.

## Results

### Participants

The participants were 15 parents (11 mothers and 4 fathers) with mental illness who were under the care of outpatient mental health services in an NHS Foundation Trust. Participants had a range of diagnoses including psychotic disorders, mood disorders, anxiety disorders, and personality disorders, with some participants having multiple co-occurring diagnoses (Table 2).

**Table 2:** Mental health diagnoses of the 15 participants interviewed in the study.

Diagnosis	Number
Attention deficit hyperactivity disorder (ADHD)	2
Anxiety disorder	3
Bipolar disorder	9
Eating disorder	2
Emotionally unstable personality disorder (EUPD)	2
Postnatal depression	1
Postpartum psychosis	1
Post-traumatic stress disorder (PTSD)	2
Recurrent depressive disorder	3
Schizoaffective disorder	1

The mean duration of time since first onset of mental illness (excluding ADHD) was 16 years (range: 1–42 years). Nine participants had their first onset of mental illness after becoming a parent, with most of these (*n* = 6) having their first onset of mental illness prior to their eldest child turning 5 years old. The mean duration of parenthood was 15 years (range 3–41 years) and the mean number of children per participant was 2 (range: 1–5).

### Themes

Results are discussed under the following themes regarding parental disclosure of mental illness (Table 3).

**Table 3:** Themes and subthemes regarding parental disclosure of mental illness.

Theme	Subtheme
1. Factors that affect what children are told about parental mental illness	1.1 Age 1.2 Concerns about the impact of the information on the child and the family 1.3 Disease specific disclosure
2. Perceived benefits of talking to children about parental mental illness	
3. Experiences of ‘who’ talks to children about parental mental illness	
4. Role of healthcare professionals in supporting families to talk about parental mental illness	

Most participants reported that they, or their partner, had acknowledged with their children that they had an illness or sometimes ‘*did not feel well*’, but there was wide variation in the extent to which children had been informed about the type and severity of the illness. These ranged from completely concealing all (or some aspects) of their mental illness, to describing some of their symptoms, or using the diagnostic name of their illness. For some parents, their explanations had been prompted by the visibility of their symptoms, medication regimen or hospital admission, but there remained extensive variability in the level of detail provided.

### Factors that affect what children are told about parental mental illness

#### Age

Many parents described a gradual approach to how their children were told about their illness which evolved organically in response to their child’s age and level of understanding.

*“It tends to be open conversations when you are sitting in the car or going for a walk… I do not think that we could have a sit-down with him and say ‘right on to the agenda we are gonna talk about that.’”* [Participant 9].

A few parents reported telling their children about the diagnosis or hospital admissions from a young age. One participant reflected: *“We’ve always told him, we have been very open right from, you know, that Mum’s bipolar. She gets manic or she’s not sleeping, so we have never hidden it from him.”* [Participant 9]; However, for most parents the amount of information shared with their children was described as increasing with their children’s age. Many parents of younger children reported using general descriptions of illness that focused on symptoms such as “*I’m unwell*” or “*I’m tired”* [children aged 4 and 2 years] *or “need quiet time”* [child aged 6 years]. Children were reported to be aware of their parent’s medication, which was described to them as “*helping me feel better*” [child aged 6 years].

*“And, you know, my son... every question is ‘why?’ You know, ‘why?’ And with that, with the... with the kind of mummy’s medication, I just, I... I’ve always had this...I do not know. I’ve always said, ‘well, you know, you take Calpol sometimes, do you not? And this is mummy’s Calpol.’”* [Participant 14; Children age 5 and 2].

Children were also told that their parent was in hospital, but not explicitly for mental health difficulties. Several parents gave explanations which broadly referenced their head or brain, such as “*Mummy’s brain is not well at the moment”* [Participant 11; Child aged 8 years] and “*Mummy’s a bit poorly, my head’s a bit poorly so I have to go to a special place to help with my head.”* [Participant 15; Child aged 5 years].

Parents described their belief that young children would not be able to understand the specifics of an illness, for example.

*“I think it’s important that we speak to them, it’s important when we think they are ready, [Name] is way too young definitely at 6...he is so into everything so he will not get it.”* [Participant 5].

Some parents reported talking “*honestly*” to their children about their mental illness but only when they were/are adolescents or young adults. This appeared to relate to parents’ perception that their child was ‘old enough’ to understand and cope with information about mental illness; *“I knew it was the right time to sort of talk about it, it was almost impossible to communicate until she was mature enough......So when she reached mid-teens, so like 15, I became a bit more open.”* [Participant 16]. Another participant anticipated telling their child *“when he is maybe 18 plus...adults or young people, maybe then they can manage it, maybe they can understand.”* [Participant 12].

#### Concerns about the impact of the information on the child and the family

Many parents expressed concerns about the potential impact of learning about their mental health difficulties on their children. These worries included the child feeling afraid or blaming themselves; *“I worry that he’s going to think he’s caused me to become sad.... I just do not want him to think that things are his fault or responsibility.”* [Participant 14]. Parents were also concerned that telling children about the illness might leave them feeling in some way responsible for taking care of their parent (either in the past or future). One parent reflected *“I want to be a parent to [Name of child] not parented by her because she feels she has to care for me in some way.”* [Participant 3], with another explaining their decision not to tell their children about the illness because *“I do not want them to start walking on egg shells or sensor or filter or treat me any differently to what they did before I got ill”* [Participant 10]. Some parents reported that they had limited explanations about their illness in order to protect their children from confusion or distress;

*“I was in hospital but did not clarify what kind of hospital. I have not told him proper. I do not want to make him upset and be like... ‘I was here’, because he’s gonna just think ‘why did not you call me’ or ‘I could have helped’.”* [Participant 5].

Some parents reflected that their uncertainty about how to answer their children’s possible questions about their illness inhibited them from talking to their children. Others reported that the stigma of their diagnosis had influenced their decision about sharing information with their child; parents were fearful that their child might tell other people about the illness. *“He comes home with stories from the school playground about things that have gone on in their homes...I do not want him to say something at school and everyone in the local network to know what I’ve got.”* [Participant 14].

Parents who felt they were currently ‘well’ expressed a strong desire to focus on normality rather than re-visiting a time when they were unwell or symptomatic. One parent reflected *“Probably because I’m in a better place now. I do not want to revisit it with him.”* [Participant 5]. Others attributed a reluctance to talk to their children based on a hope that the conversation might not be needed; “And *also, I think because I have this secret hope that I might just get better … then I sort of think, ‘Well, maybe it’s not necessary’.”* [Participant 14].

Some parents reported a fear that their children might ‘*copy parental behavior*’ if they were aware of certain aspects of their parent’s mental illness. This was specifically highlighted by parents with lived experience of self-harm and eating disorders, for example *“I do not want him to think that [self-harm] that’s a healthy way for an adult or anyone to deal with their emotions.”* [Participant 13] with another parent reporting *“They have no idea I had an eating disorder … and that’s because I can see it in [daughter] and I do not want to put ideas in her head.”* [Participant 1].

#### Disease specific disclosure

Participants had different approaches to sharing some, or all, of their psychiatric diagnoses with children. Parents reported talking to children about their experiences of anxiety, mania, psychosis, PTSD and ADHD. The reasons for these choices often reflected parents’ beliefs that certain aspects of their illness were easier to talk about or explain to children. For example, one parent explained their decision to share some, but not all, of their diagnoses as *“I’ve stuck with the concept of worrying [name given to anxiety]. I do not think they are ready for the concept of the depression though.”* [Participant 4].

Others gave detailed rationales about how they had approached these conversations with their children, which included the use of humor to try and minimize the emotional impact of the illness, such as *“We joke about it now, how I had a reduced attention span and how I would take risks that were not sensible...she thinks it’s hilarious and we talk about it quite a lot.”* [Participant 8]. This approach was echoed by a parent describing conversations about her behavior during a psychotic episode. *“I’ve talked about it as like ‘ohh the silly things I did when I was psychotic’...we have laughed about some situations with him and made light of it... so we would be laughing about it: ‘Oh yeah, the time that mummy thought this or that, that funny place where mummy jumped out of the car and went bonkers.’”* [Participant 9].

Some parents had found books and films a useful reference point to help their children understand parental mental illness.

*“I say ‘it’s like in films when the army people have flashbacks all the time.’ In the films they usually show them getting help, probably because they are soldiers... and they are messed up.... and PTSD [has] a reason... this created PTSD or that created PTSD. So it’s easier to explain and [give] the reason.”* [Participant 5].

Symptoms or diagnoses that were deliberately not shared with children included self-harm, suicidal ideation, depression and eating disorders. Participants reported that these would be too frightening or upsetting for children, with one parent explaining *“We’ve not told them [details of suicide attempt] … the train track is just shocking and you get a very graphic image and it’s so final and we just did not want to say that.*” [Participant 1].

Many parents chose not to tell children about their depression, offering a range of different reasons for this decision. Some parents felt it was “*easier to hide*” than other aspects of their mental illness, while others avoided talking about depression as they felt it was too painful or conflicted with their perceived role within the family. For example, one parent reported *“I’m the protector... you do not want to see your protector kind of fall.... you wanna see clinking armor, not a weakness because they are looking at you for support”* [Participant 5].

Consistent with parents’ use of humor when talking about some diagnoses to minimize their emotional impact (described above), the absence of such opportunities inhibited parents from talking about other aspects of mental illness:

*“I think with the pure depression, I think I’m just more ashamed of it, and it’s harder to explain. So I think that’s probably why I have avoided it. So there’s not a funny angle or anything like that, it’s too, too bleak. So I think that’s probably why I have not done it”* [Participant 8].

Others deliberately avoided using the name of their mental illness to so their child would not “*read up on it.,”* reflecting parental fears about the impact of this information on the child.

### Perceived benefits of talking to children about parental mental illness

Participants identified a number of perceived benefits about sharing information about their mental illness with children. Some expressed a belief that *not* being honest with their children about the illness would create more anxiety, for example.

*“There’s nothing to hide, I think when you hide things from children the ideas can build up in their own mind, its scarier that adults are not explaining...it must be terrible that your parent won’t tell you.”* [Participant 6].

Others focused on their children’s increased vulnerability to developing a mental illness, and therefore the importance of the child recognizing their own symptoms in the future or avoiding additional risk factors.

*“So with both of them, we have had talks about, you know, do not take drugs, the brains in our family are too fragile. And I’m particularly trying to get that across to [name] because I think she’d be the kind of one who’d like to show that she’s trying this or having drink before the others. I can just see her doing that kind of thing.”* [Participant 1].

Other parents reflected that they talked about their own mental health when one of their children was experiencing emotional distress or “*going through a difficult time*” (e.g. bullying). These participants felt that this provided an opportunity to show an understanding of their child’s emotional difficulties and that drawing on their own experiences felt helpful in supporting their child.

*“When [child] says something about being down, I will open up and say like ‘you know I’ve been through this. I can help you and stuff.’ It’s helped me be a better Dad with him. And he has opened up to me more than his Mum because he knows that I’ve been through stuff and knows that I’m speaking the truth about stuff.”* [Participant 5].

Some, although not all, of the parents who had concerns about the impact of sharing information on their children were also able to recognize the benefit of talking to them about their mental illness in certain circumstances, specifically if their children were experiencing similar difficulties.

*“Yes, if I see if I see [name] begins to develop an eating disorder, that’s when I’ll start telling her and ways to cope. But I will not say anything otherwise so I’m not introducing anything unnecessarily. They do not need to know that, unless they do need to”* [Participant 1].

### Experiences of ‘who’ talks to children about parental mental illness

Most participants implicitly indicated a sense of shared responsibility for talking with children, through the frequent use of “we” when discussing their decisions and rationale for communicating (or not communicating). In addition, many explicitly acknowledged the important role of the ‘well’ parent, reporting that they had been too unwell to talk to their children around the time of a hospital admission and so had been reliant on the ‘well’ parent to explain what was happening. Many said if children did have questions, they were more likely to ask the ‘well’ parent as they were physically ‘*around more*’, with some expressing uncertainty about how these questions had been answered or discussed. One participant acknowledged *“… she asked the name and about the illness to my husband and not to me.”* [Participant 11].

Several parents reflected that the crisis nature of their hospital admission meant it had been difficult to consider their children’s needs or think carefully about what they had told their children.

*“I think we were so much scrambling around trying to survive that I just do not think we thought of them enough. I was just so consumed with, like getting through the day. I do not think I spoke to them that much about it at the beginning.”* [Participant 8].

Some parents were aware that other people in their family and social network were talking about their mental illness. They felt concerned about what these individuals understood about their illness and how this could impact on explanations in response to their children’s questions. As a consequence, a few parents highlighted the importance of consistent messaging about their illness within families.

*“.... I also think it’s important that other people he’s regularly in contact with are like we are all on the same page.... they spend time with him and my parents and like to make sure that we are kind of all on the same wavelength of how we talk about it and in case he asked questions….”* [Participant 13].

The importance of consistency in communication about mental illness was also raised by participants who were separated from their children’s ‘well’ parent. Some participants reflected that because of the breakdown in their relationship the ‘well’ parent did not always receive a lot of information about the mental illness and thus could not provide accurate explanations to the child. One participant highlighted the negative impact that this had had on his daughter: *“My ex-wife was very upset cause she wasn’t told I had gone into hospital....my daughter’s understanding has been very clouded by her mum because her mum is scared of it [bipolar]”* [Participant 3].

### Role of healthcare professionals in supporting families to talk about parental mental illness

All participants reported that healthcare professionals had **never** asked them what their children knew about their mental illness or offered specific guidance on how to have these conversations. Participants consistently reported *“No, no one’s ever mentioned the children.”* [Participant 8]. However, parents also expressed the importance of recognizing their role as parents and the impact of the illness on their children:

*“It’s been at the forefront of my mind, but no one else’s.” [Participant 1]*.

Crucially, all participants said they would have welcomed any help and support on having conversations with their children.

*“...I think it would [help] actually, I obviously have some lived experience from my friends, but it’s definitely missing to have the professional inputs...”* [Participant 9].

Participants suggested healthcare professionals could provide guidelines and strategies on how to have these conversations with their children; these included age-appropriate resources, a helpline which children could access independently, and a ‘space’ for families to talk together. Some participants wanted professionals to educate them about their diagnosis, which would in turn help them then talk to their children about the illness. Many parents were concerned about “getting it right” and consequently wanted guidance about what they should, and should not say about their illness. One participant requested *“If there were any books.... I’d be keen to read some to them [the children], make sure I’m doing it right with the kids.”* [Participant 4]. Other parents wanted reassurance from a professional to provide them with the evidence of the benefits of why children should be told about parental mental illness and how other families had managed this situation:

*“If when I was first diagnosed and I was being given advice on how to address it with my children, (how to talk about it at home, about openness and things like that), it would be knowing that others had experience where it had a really positive outcome for the children.”* [Participant 6].

Participants again highlighted the importance of ensuring the ‘well’ parent was included in such discussions, particularly when parents were separated.

*“Just some guidance for parents would be really useful. Both for the sufferers of mental health conditions, but also their partners. Uh, probably more so for the partner because they are the one that has to do the explaining if you go into hospital.”* [Participant 3].

*“I wasn’t well enough to realize it [mental illness] was actually impacting the children. You just do not realize you are that much switched off.... you cannot really communicate with them properly which is why you need a resource or something to look at...”* [Participant 15].

Two participants reported that a healthcare professional had offered to talk directly to their children about the illness but this offer was not taken up by the families as they felt the professional was not the right person to do this task. One parent reflected “..*.mental health people get involved and they do not know your children, the kids would think ‘who’s this stranger talking to me about my mother?’”* [Participant 10]. Another parent indicated that they felt guidance from healthcare professionals about talking to children, would be valuable, but believed parents should retain autonomy about how to use this information *“I think it would have been a helpful conversation if someone had said ‘what are you doing about your children? Have you explained to them what’s going on?’ But I think it is definitely my decision about how I do.”* [Participant 8].

## Discussion

This study aimed to explore parents’ perspectives and experiences of disclosing information to their children about their mental illness, the assistance provided by clinical teams in facilitating communication with their children, and aspects that would have been beneficial for them in the communication process.

Empowering parents to have honest conversations with their children about mental illness could potentially reduce stigma and promote well-being for the whole family. Effective communication about parental mental illness helps children understand their parent’s symptoms (Garley et al., 1997), including unusual, unpredictable or upsetting behavior (Van Parys et al., 2015), which can bring a sense of relief and mitigate feelings of responsibility and blame (Reupert and Maybery, 2010; Backer et al., 2017). Furthermore, when families can talk together about challenges and distressing experiences, it helps strengthen their family connectedness (Pihkala et al., 2012), allows children to access social and emotional support (Cudjoe and Chiu, 2020), and in turn enhances resilience (Focht-Birkerts and Beardslee, 2000). Parents discussed that by disclosing their vulnerability and the signs of mental illness, they could help their children to recognize these symptoms in themselves or their peers. This knowledge could facilitate early intervention for any potential mental health concerns, improving long-term outcomes. In addition, parents felt that their own experiences of emotional distress and challenges enabled them to relate to their own children when they were going through similar situations.

Strikingly all participants reported that they had not been given any guidance from professionals involved in their care about talking to their children. Consequently, their decisions were based on personal judgment about what ‘they thought best’, often during periods of immense family stress due to the illness itself. Importantly, most parents reported that they would welcome specific support from their healthcare team about communication with children; this included both a discussion about the potential benefits of talking to children and reassurance that this would not lead their children to ‘copy’ parental behavior (such as self-harm). This aligns with existing research reporting that parents want ongoing support for themselves and their children (Dunn et al., 2020), including how to structure these conversations (Wolpert et al., 2015; Chen et al., 2021). Children themselves have expressed a need for information about their parent’s condition to help them understand illness-related behavior (Mordoch, 2010) with a preference to receive this from healthcare professionals (Grove et al., 2016).

Parents often relied on their partners or ex-partners to take the lead role in such conversations, particularly at times when they had become seriously unwell. This highlights the necessity for clinical teams to maintain comprehensive and up-to-date information about patients’ family circumstances, particularly when parents may be estranged or separated. It also requires clear documentation regarding patients’ wishes about who needs to be informed should they experience a significant deterioration in their mental health. Recording families’ preferred words in clinical notes to talk about the illness with children could be one way to ensure consistency in communication across different caregivers.

Whilst mental healthcare professionals acknowledge their role in identifying children in affected families, the primary focus is often on safeguarding, rather than children’s experience of illness within the family (Radley et al., 2021; Dalton et al., 2024). Previous research has indicated that some mental healthcare professionals hesitate to initiate discussions about communication with children, believing this would not be welcomed by patients and might distress their patient, or negatively impact on their therapeutic relationship (Johansson et al., 2014; Krumm et al., 2019; Dalton et al., 2024). However, family members and carers affected by mental illness emphasize the importance of identifying and acknowledging their relationships and responsibilities to children (Harries et al., 2023). Sharing families’ desire for recognition and support with clinical staff is an essential foundation on which to build a strategy for promoting communication with children about mental illness. This approach must address the documented obstacles for professionals, including both a lack of specific information about what children of different ages understand about mental illness, and training in how to undertake this task (Reupert and Maybery, 2010; Cudjoe and Chiu, 2020; Dalton et al., 2024).

The importance of using age-appropriate communication strategies and language when talking to children about parental mental illness is widely recognized (e.g., Reupert and Maybery, 2010; Tabak et al., 2016; Cudjoe and Chiu, 2020). However, research on children’s evolving understanding of illness has predominantly focused on physical illness, with mental illness remaining a neglected area of academic study (Mueller et al., 2016; Georgakakou-Koutsonikou and Williams, 2017). Whilst some evidence suggests that children’s factual accuracy in differentiating between physical and mental illness improves with age, this work has been limited to their understanding of illness causality, curability and time course (Fox et al., 2010; Fox, 2020). Further research is needed to explore children’s perception of ‘illness identity’ (i.e., the label used for an illness or collection of symptoms) (Leventhal et al., 1980) at different ages and for different mental illnesses. This will ensure professionals and families use the most appropriate language when talking to children about parental mental illness, ensuring comprehension and preventing misunderstandings.

There are several evidence-based interventions to support parents in talking to children about their mental illness, including The Family Model (Falkov et al., 2020), Kidstime (Wolpert et al., 2015) and Family Talk (Mulligan et al., 2021; Furlong et al., 2024). However, these interventions have not been sustainably embedded into routine clinical services. In part, this may reflect the intensity and duration of the interventions, staffing requirements and physical space required for group sessions at a time of increasing pressure on mental health services. However, supporting families to talk with children about parental mental illness is predicated on first identifying these families. This task remains a significant challenge, with a recent survey of over 1,000 mental healthcare professionals working in the UK revealing that a quarter did not routinely identify whether patients had dependent children (Dunn et al., 2022).

### Strengths and limitations of the study

This study explored both mothers and fathers with a spectrum of diagnoses, although most patients had a diagnosis of bipolar affective disorder. The experiences of the ‘well’ parent or the children in these families were not investigated. This study complements existing work focusing on interventions aimed at improving family communication about parental mental illness by adding to our understanding of the extent and ways in which parents with mental illness currently talk about their mental illness, and their views and preferences on how they would like to be supported. This study is limited by only including the experiences of participants from one NHS trust.

### Relevance for practice

This study highlights that mental health services can play an important role in working with patients to promote effective communication about parental mental illness, and there are multiple areas which should be considered in order to improve practice in this regard. Firstly, age-appropriate resources and guidelines should be developed to equip parents with the knowledge and skills to effectively communicate with their children about mental illness. This must also include primary research to elucidate children’s understanding and use of illness ‘labels’ at different ages. Secondly, healthcare professionals should be provided with training and specific guidance on how to support families in discussing parental mental illness with children. This could involve developing resources to support healthcare professionals initiate conversations with parents, answer parents’ questions and provide families with resources to use directly with their children. Finally, there should be a focus on exploring the specific challenges and needs of other caregivers such as ‘well’ parents (particularly those who are separated or estranged) when talking about mental illness with their children. This may also require exploration of how the wider family can be recognized and supported by healthcare systems.

## Conclusion

Despite the well documented benefits of effective communication, many parents struggle with talking to their children about a mental illness due to uncertainty about how to navigate these sensitive conversations and a fear of burdening their children. Providing appropriate resources and support structures can facilitate effective communication. Professionals are key in helping empower parents to disclose their mental illness authentically and effectively. This, in turn, can foster positive mental health outcomes for both parents and children, strengthen family relationships and promote overall well-being.

## Data Availability

The datasets presented in this article are not readily available as they relate to a highly vulnerable population and include data relating to their diagnosis, number and ages of children. The participants were recruited from a single NHS trust (which could be identifiable from the affiliations of the authors) and therefore risks potential identification of the families involved. The datasets are available on reasonable request to the corresponding author. Requests to access the datasets should be directed to louise.dalton@psych.ox.ac.uk.
